# Reduced β-catenin expression affects patterning of bone primordia, but not bone maturation

**DOI:** 10.1242/bio.023572

**Published:** 2017-03-27

**Authors:** Tobias Pflug, Uyen Huynh-Do, Stefan Rudloff

**Affiliations:** Department of Clinical Research, Department of Nephrology and Hypertension, Bern University Hospital, University of Bern, Freiburgstrasse 15, Bern CH-3010, Switzerland

**Keywords:** β-catenin dosage, Canonical Wnt signaling, Bone development, Limb patterning

## Abstract

Wnt/β-catenin signaling is involved in patterning of bone primordia, but also plays an important role in the differentiation of chondrocytes and osteoblasts. During these processes the level of β-catenin must be tightly regulated. Excess β-catenin leads to conditions with increased bone mass, whereas loss of β-catenin is associated with osteoporosis or, in extreme cases, the absence of limbs. In this study, we examined skeletogenesis in mice, which retain only 25% of β-catenin. These embryos showed severe morphological abnormalities of which the lack of hindlimbs and misshaped front paws were the most striking. Surprisingly however, calcification of bone primordia occurred normally. Moreover, the Wnt-dependent regulatory network of transcription factors driving the differentiation of cartilage and bone, as well as the expression of extracellular matrix components, were preserved. These findings show that 25% β-catenin is insufficient for the correct patterning of bone primordia, but sufficient for their mineralization. Our approach helps to identify bone morphogenetic processes that can proceed normally even at low β-catenin levels, in contrast to those that require high β-catenin dosages. This information could be exploited to improve the treatment of bone diseases by fine-tuning the individual β-catenin dosage requirements.

## Abstract (precis)

**Summary:** The correct allocation and patterning of skeletal elements during development requires a higher dosage of β-catenin than the maturation and mineralization of these primordia.

## INTRODUCTION

Canonical Wnt signaling activates the transcription of β-catenin-dependent target genes during many stages of bone development and maintenance (Duan and Bonewald, 2016), whereby a cytoplasmic multi-protein complex composed of the scaffolding proteins Axin and APC (Heuberger and Birchmeier, 2010; Munemitsu et al., 1995), and the kinases CK1α and GSK3β (Behrens et al., 1998; Munemitsu et al., 1995), tightly regulates the level of β-catenin. In the absence of canonical Wnt signaling this degradation machinery continuously phosphorylates β-catenin at its N-terminal region. This marks the protein for ubiquitination and proteasomal degradation (Aberle et al., 1997) and the transcription of canonical Wnt target genes is repressed. Upon binding of a canonical Wnt ligand to the co-receptor pair Frizzled and Lrp5/6 (Heuberger and Birchmeier, 2010), the degradation machinery is disassembled and β-catenin accumulates in the cell and is subsequently translocated to the nucleus, where it transactivates canonical Wnt target genes (Cadigan and Peifer, 2009; Heuberger and Birchmeier, 2010; Roose et al., 1998). Additional players that affect the level of β-catenin during bone development and repair include the extracellular Wnt inhibitors Dickkopf and Sclerostin (Florio et al., 2016; Ke et al., 2012; Qiang et al., 2008).

Early during embryonic development Wnt/β-catenin signaling plays crucial roles in determining the future body axes, and maintaining gastrulation and mesoderm production (Hikasa and Sokol, 2013; Holstein, 2012). During later stages, Wnt signals are important for induction, growth and organization of the future limbs (Kengaku et al., 1998). Hereby, reciprocal signals between mesenchymal and ectodermal cells promote the formation of the limb organizer, called the apical ectodermal ridge (AER) (Barrow et al., 2003; McQueeney et al., 2002; Narita et al., 2007). The AER promotes the proliferation of the underlying mesenchymal cells via Fgf signaling and induces the zone of polarizing activity (ZPA) at the posterior side of the growing limb bud. Limb patterning involves multiple signaling protein gradients (Wnt, Fgf and Shh), originating from either AER or ZPA, that in turn affect the expression of different Hox genes (Wellik and Capecchi, 2003; Capdevila and Izpisúa Belmonte, 2001; Chiang et al., 2001). The individual bones of stylopodium (humerus or femur), zeugopodium (radius/ulna or tibia/fibula) and autopodium (hand or foot) are initially set up as aggregates of tightly packed mesenchymal cells (Hall and Miyake, 2000). These mesenchymal condensations differentiate into cartilage and are later replaced by bone via endochondral osteogenesis. The orderly progression of these processes again crucially depends on the timely activation of Wnt/β-catenin signaling in the respective cell types (Lu et al., 2013; Tamamura et al., 2005). The molecular mechanisms underlying bone formation are highly complex and involve the activation of mutually exclusive transcription factors and extracellular matrix genes in chondrocytes and osteoblasts. As the master regulator of chondrogenesis, Sox9 drives not only the expression of cartilage-specific collagen type II and Aggrecan, but also maintains the proliferation of chondrocytes and prohibits their differentiation into osteoblasts and thus ossification (Lefebvre and de Crombrugghe, 1998; Dy et al., 2012; Hattori et al., 2010). The activity of Sox9 is itself regulated by several upstream signals, of which canonical Wnt signaling exerts an inhibiting effect. Lastly, Sox9 inhibits the expression of Runx2, a key transcription factor for bone development (Liu and Lee, 2013). Conversely, activation of canonical Wnt signaling in pre-osteoblasts triggers the expression of Runx2 and other bone-promoting factors such as Sp7 or Atf4 (Huang et al., 2007; Yang and Karsenty, 2004). Their importance for osteoblast differentiation has been shown in knock-out mice or overexpression studies, which result in loss of bone formation or ectopic ossification, respectively (Komori et al., 1997; Ueta et al., 2001). Furthermore, these transcription factors activate genes like type I collagen alpha 1, osteocalcin or bone sialoprotein that are characteristic for the extracellular matrix of bone (Ducy et al., 1997; Kern et al., 2001; Ganss et al., 1999).

It is therefore not surprising that many human genetic diseases are associated with mutations that cause a loss of function (LOF) or a gain of function (GOF) of different genes of the Wnt signaling pathway. GOF mutations in both mice and humans lead to unusually high bone mass (Regard et al., 2012), while LOF mutations are usually associated with low bone mass. One of the most extreme LOF mutations is the Tetra-amelia syndrome. It is caused by mutations in WNT3, which leads to the complete loss of the formation of all four limbs (Niemann et al., 2004). In this study, we restricted β-catenin expression during bone development without disturbing the machinery of the Wnt signaling pathway. To this end, we conditionally expressed *β-catenin* only from the *ROSA26* locus (Rudloff and Kemler, 2012) in all tissues caudal to the heart (Hierholzer and Kemler, 2009). We found that the morphogenesis of the distal forelimbs, hindlimbs and the tail was disturbed. We further showed that the expression of genes underlying the patterning process in the forelimb autopodia was severely disturbed. Yet, the formation of mineralized matrix in these bone anlagen appeared to proceed normally. More surprisingly, we could show that the underlying molecular machinery that controls the differentiation of chondrocytes and osteoblasts was preserved in *β-catenin* knockdown mice. Thus, low β-catenin expression levels are insufficient for correct patterning of the skeleton, but adequate for the maturation of skeletal primordia.

## RESULTS

### Knockdown of *β-catenin* leads to developmental defects in the caudal embryo

**Fig. 1.**
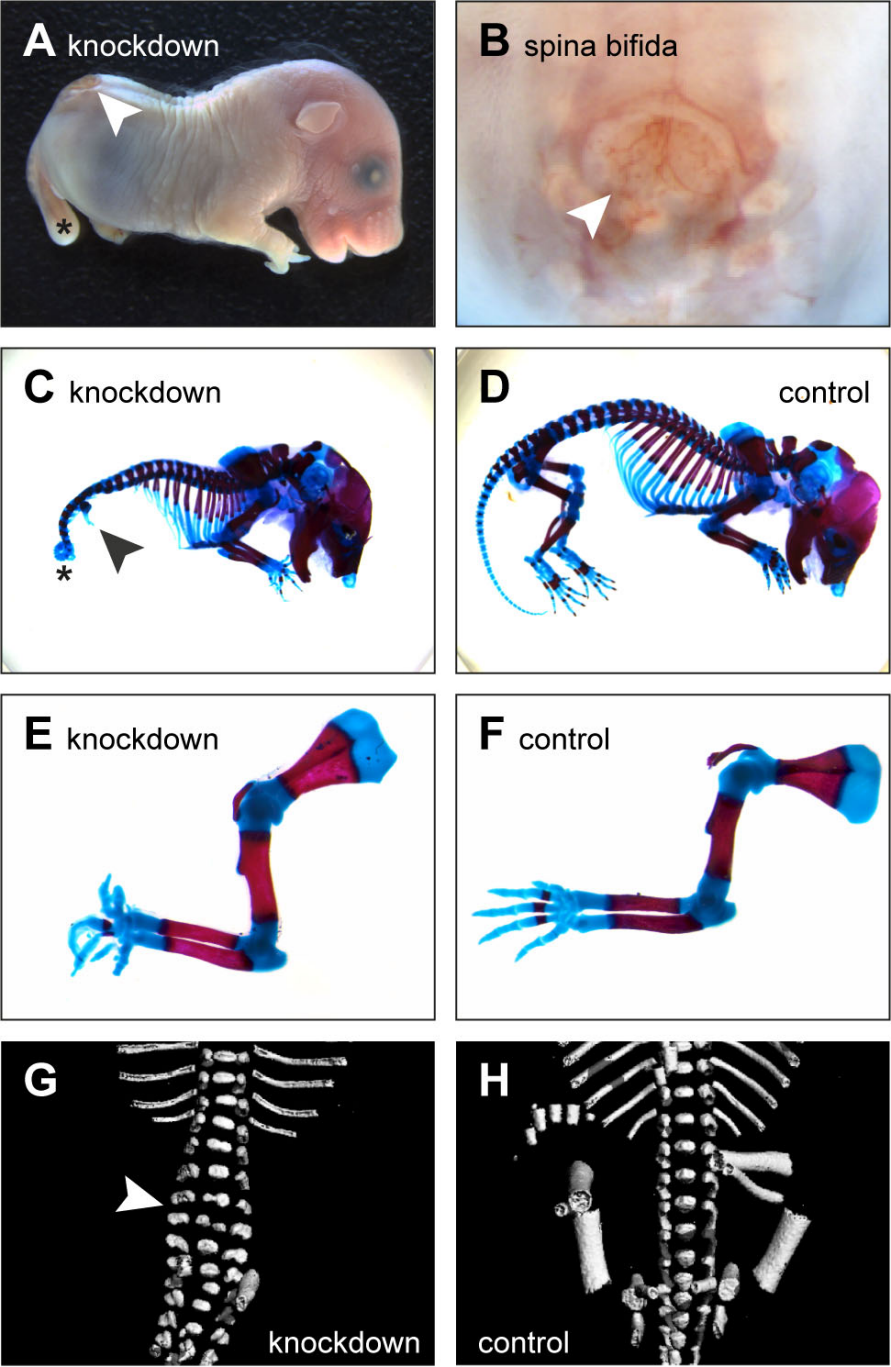
**Phenotype of β-catenin knockdown embryos.** (A) Representative E18.5 *β-catenin* knockdown embryo showing malformed forelimb digits, a misshaped tail (asterisk) and an open back (spina bifida, white arrowhead). (B) Close-up image of spina bifida (white arrowhead). (C,D) Whole mount skeletal preparation of a knockdown (C) and a control (D) E18.5 embryo. In the knockdown, the rudimentary pelvic bones (black arrowhead) and the curled tail (asterisk) are indicated. (E,F) Skeletal staining of isolated knockdown (E) and control (F) forelimbs showing similar proximal structures (scapulae, stylopodia and zeugopodia), but severely misshaped digits in the mutant compared to the control. (G,H) Frontal micro CT scans of knockdown (G) and control (H) E18.5 embryos showing split lumbar vertebrae (white arrowhead in G), absence of hindlimbs and almost horizontally protruding ribs in the mutant.

Expression of *Cdx1::Cre* mediates recombination in the three germ layers of the primitive streak region throughout the posterior embryo, caudal to the heart at mid-gastrulation (Hierholzer and Kemler, 2009). Thus, in (*Cdx1::Cre*)(*β-catenin flox/flox*)(*ROSA26::β-catenin*) mice, the endogenous *β-catenin* alleles are deleted and replaced by the *ROSA26::β-catenin* transgene. Homozygous expression of *ROSA26::β-catenin* leads to a β-catenin expression level of 25% compared to wild-type mice (Rudloff and Kemler, 2012). These mice, which exhibit severe malformations, are referred to as knockdown or mutant embryos throughout the text. For a better understanding of where the *Cdx1::Cre* mediated manipulation is active in our mutant mice, we performed histological and genetic analyses. Sagittal sections of embryonic day (E)18.5 knockdown embryos revealed that internal organs (e.g. heart, intestine, kidneys, liver, lungs and pancreas) were present, excluding a general defect during early gastrulation (Fig. S1). Furthermore, we isolated DNA from these organs, as well as from brain, skin (head and abdomen), abdominal muscles and tail. Subsequent genotyping for endogenous *β-catenin* revealed that all tissues of foregut endodermal origin (i.e. small intestine, liver, lung and pancreas), anterior mesoderm-derived structures such as heart and spleen, and ectodermal organs like brain and skin from the head region still carried a floxed *β-catenin* allele (324 bp band in Fig. S1). This means that Cre recombination had not taken place in these tissues. On the other hand, all posterior mesoderm-derived tissues (e.g. kidney, abdominal muscles and tail) and skin isolated from the caudal half of the embryo lacked the floxed allele and only showed the deleted *β-catenin* gene product (500 bp band in Fig. S1). Taken together, Cre-mediated recombination in our knockdown embryos took place in all mesodermal and ectodermal tissues that are derived from the posterior embryo at midgastrulation, including the fore- and hindlimb primordia. Phenotypically, mutant embryos showed major developmental defects with increasing severity in the caudal half of the embryo (hindlimbs and tail), as well as in the distal parts of the forelimbs (Fig. 1; Fig. S2). In more detail, most mutant embryos did not have any hindlimb structures (Fig. 1A,C and G), displayed a spina bifida (white arrowhead in Fig. 1A,B), an unusually curled tail (asterisk in Fig. 1A,C and Fig. S2A) and only rudimentary pelvic bones (black arrowhead in Fig. 1C). In very few embryos, truncated hindlimbs were present (black arrowhead in Fig. S2A). Forelimbs were always developed; however, their appearance was abnormal, and the digits showed severe malformations (Fig. 1A). To gain more insight into the skeletal changes, we performed Alcian Blue (cartilage) and Alizarin Red (calcified bone) staining on whole mount embryos (Fig. 1C,D; Fig. S2A) and isolated forelimbs (Fig. 1E,F; Fig. S2B-D). Shape and size of the skeletal elements of forelimb zeugopodia and stylopodia seemed not to be altered between control (Fig. 1F) and *β-catenin* knockdown embryos (Fig. 1E; Fig. S2B-D). In rare cases, the forelimb zeugopodium consisted of only one bone (Fig. S2B). Conversely, the autopodia of *β-catenin* knockdown embryos always showed severe morphological alterations, with carpals, metacarpals and phalanges exhibiting irregular proportions and orientations (Fig. 1E; Fig. S2B-D). Hereby, the number of digits could be either increased (Fig. 1E) or reduced (Fig. S2B,C) and in some cases fused phalanges were detected (Fig. S2D). Micro CT scans of control and mutant embryos (Fig. 1G,H; Fig. S2E,F) confirmed the findings of the skeletal staining. Moreover, in knockdown embryos, broadened, split lumbar vertebrae were found (white arrowhead in Fig. 1G), providing a morphological explanation for the spina bifida. Furthermore, in mutant embryos the ribs protruded at a flatter angle compared to controls and the intercostal distance seemed to be increased (Fig. 1G,H; Fig. S2E,F). Interestingly, there were only 11 instead of the expected 12 rib pairs present in the micro CT-scanned knockdown embryo (compare Fig. S2E to F).

### Altered expression of limb patterning genes in *β-catenin* knockdown embryos

**Fig. 2.**
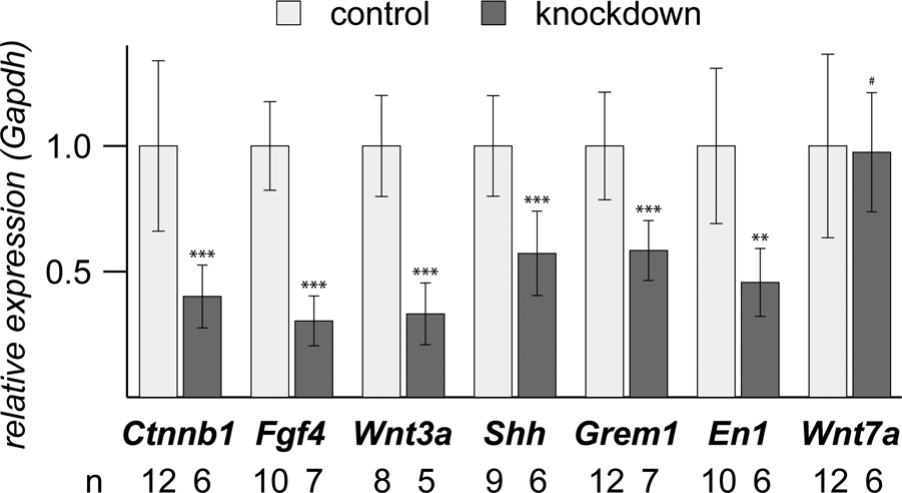
**Transcripts of limb patterning genes are down regulated in *β-catenin* knockdown embryos.** *β-catenin* (*Ctnnb1*) mRNA expression is reduced to about 40% in knockdown embryos compared to controls. The mRNA expression levels of *Wnt3a*, *Fgf4*, *Shh* and *Grem1*, which form a regulatory loop maintaining AER and ZPA, are also significantly decreased. Furthermore, the mRNA expression of *En1*, which plays a role in AER formation and ventralization, is also significantly reduced. On the other hand, the mRNA expression level of *Wnt7a* is not changed between controls and *β-catenin* knockdown embryos. Unpaired, two-tailed *t*-tests adjusted for similar or different variances; mean±s.d. is shown; *n*, number of samples included in each analysis; each sample was analyzed in duplicate; #, not significant; **P<0.01; ***P<0.001.

The striking morphological changes of forelimb autopodia upon knockdown of *β-catenin* triggered us to analyze the expression of genes that are involved in the patterning of these structures. Two major signaling centers, the ZPA and the AER, control the morphogenesis of a limb. Hereby, Wnt3a stimulates the release of Fgf4 and Fgf8 from the AER. The Fgfs then induce the ZPA on the posterior end to produce Shh, which via Gremlin1 (Grem1) maintains Fgf expression in the AER. The Shh gradient further governs anterior-posterior patterning. Wnt7a and En1 secreted from the dorsal and ventral ectoderm, respectively, regulate dorsal-ventral patterning. We found in our analysis of E17.5 forelimb autopodia that the mRNA expression levels of all above mentioned genes were significantly reduced in *β-catenin* knockdown embryos except *Wnt7a*, whose expression was unchanged (Fig. 2). We could not detect *Fgf8* expression in our samples, which might be due to the already advanced development of the autopodia used in our analysis. In summary, our findings show that anterior-posterior and dorsal-ventral patterning is altered in *β-catenin* knockdown embryos.

### Normal calcification of proximal forelimb bone primordia in *β-catenin* knockdown embryos

**Fig. 3.**
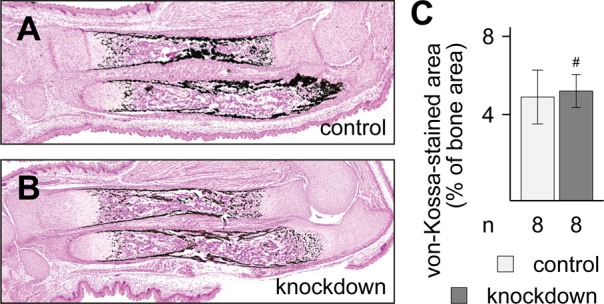
**Calcification of zeugopodial bones is unchanged in *β-catenin* knockdown embryos.** (A,B) Representative von-Kossa-stained sections show comparable areas of calcification of ulna and radius in control (A) and *β-catenin* knockdown embryos (B). (C) Quantification of the von-Kossa stained areas. No significant difference was detected (*P*=0.643). Unpaired, two-tailed *t*-test; mean±s.d. is shown; *n*, number of samples included in each analysis; #, not significant.

Apart from the morphological changes of the forelimb autopodia, we could not detect differences in the morphology and mineralization of stylopodia and zeugopodia between control and *β-catenin* knockdown embryos in the whole mount skeletal preparation. Therefore, we decided to analyze ulna and radius in more detail histologically and histomorphometrically. To this end, forelimbs were collected at E18.5 and stained for calcification using the von Kossa method (Fig. 3A,B). Histomorphometrical quantification of the von Kossa stained area of zeugopodial bones revealed no significant difference between controls and knockdowns (Fig. 3C), showing that the ossification of ulna and radius was not disturbed in *β-catenin* knockdown embryos.

### Primary *β-catenin* knockdown rib chondrocytes behave normally upon stimulation of canonical Wnt signaling

**Fig. 4.**
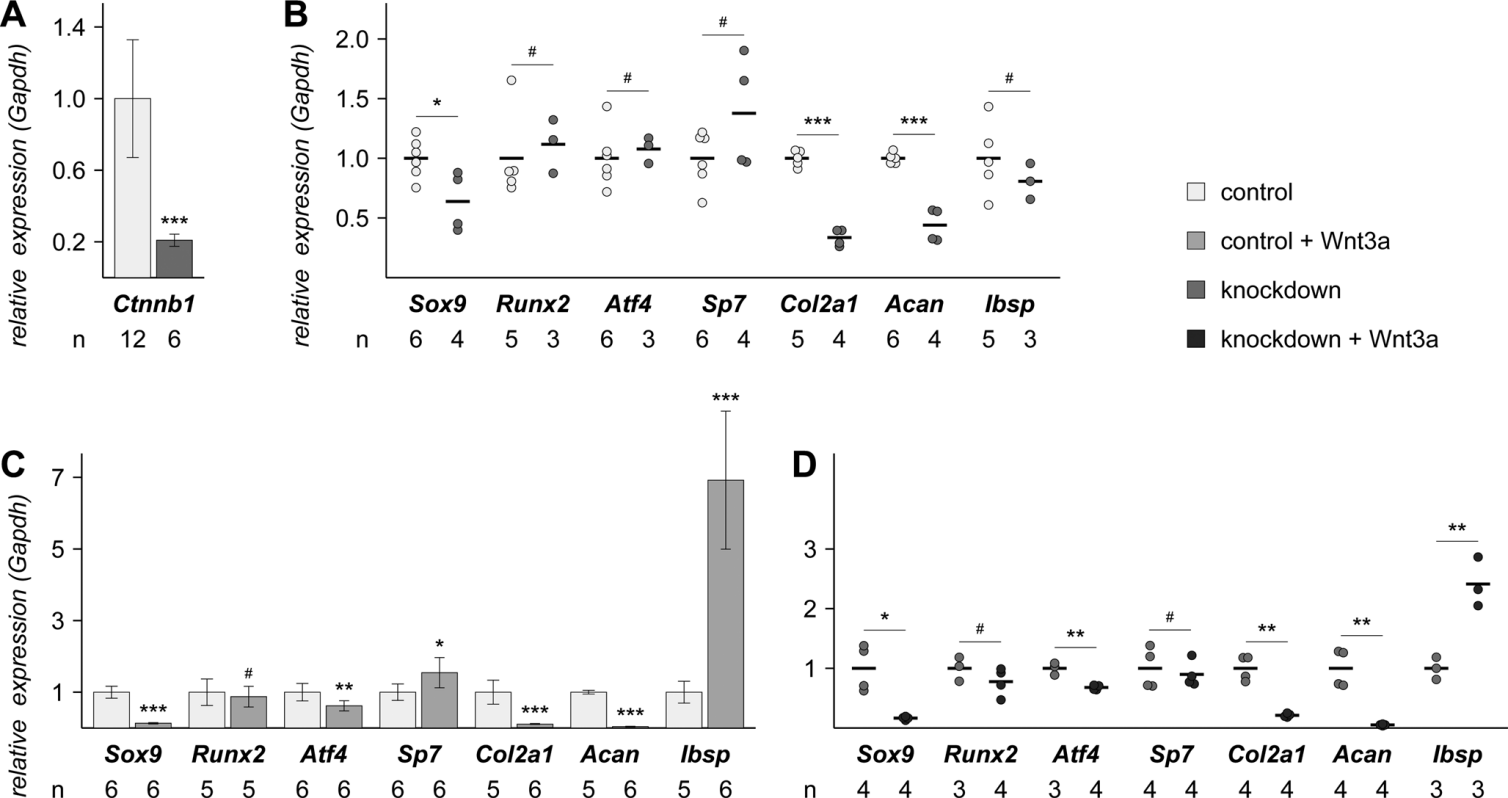
**Knockdown and control primary chondrocytes respond similarly to the stimulation of canonical Wnt with reduced mRNA expression of cartilage-specific genes and increased mRNA expression of bone extracellular matrix genes.** (A) The mRNA expression of *β-catenin* (*Ctnnb1*) is significantly reduced to 20% in primary knockdown chondrocytes, confirming the usability of the mouse model. (B) Baseline mRNA expression of bone or cartilage-specific transcription factors and extracellular matrix components. The mRNA expression of the cartilage-specific genes *Sox9*, *Col2a1* and *Acan* (Aggrecan) is reduced in knockdown cells. Transcript levels of genes important for bone differentiation (*Runx2*, *Atf4*, *Sp7* and *Ibsp*) are equally expressed in both cell types. (C,D) Stimulation of canonical Wnt signaling leads to a profound downregulation of the mRNA of genes important for cartilage in control (C) and knockdown cells (D). *Runx2* shows similar mRNA expression levels upon Wnt3a treatment in both cell types, whereas the mRNA expression of *Atf4* is reduced to 65% in both cell types. *Sp7* transcript levels are slightly up regulated in control cells (C), but unchanged in knockdown cells (D). Strikingly, the expression of the bone extracellular matrix component *Ibsp* is significantly up regulated in both cell types. Unpaired, two-tailed *t*-tests adjusted for similar or different variances; A,C show mean±s.d.; B,D show mean and individual data points; *n*, number of primary chondrocyte cell lines included in each analysis; each cell line was analyzed in duplicate; #, not significant; *P<0.05; **P<0.01; ***P<0.001.

Since the calcification of bone primordia seemed to be intact in *β-catenin* knockdown embryos, we wondered whether the regulatory network underlying bone development was affected by the knockdown of *β-catenin*. To this end, we isolated primary rib chondrocytes and analyzed the expression of several genes with a role in cartilage and bone development. Genes of interest were transcription factors that are influenced by Wnt signaling in cartilage (*Sox9*) or bone (*Runx2*, *Sp7* and *Atf4*) and extracellular matrix proteins specific for cartilage (type II collagen alpha 1–*Col2a1* and Aggrecan–*Acan*) or bone (integrin-binding sialoprotein–*Ibsp*). Cultures of primary rib chondrocytes derived from control or *β-catenin* knockdown embryos showed no morphological differences (Fig. S3) despite the reduction of *β-catenin* (*Ctnnb1*) mRNA expression to 21% in knockdown cells (Fig. 4A). In unstimulated cells, the mRNA expression levels of the cartilage-specific genes *Sox9*, *Col2a1* and *Acan* were significantly reduced in *β-catenin* knockdown cells, whereas the transcripts of the bone-specific genes *Runx2*, *Sp7*, *Atf4* and *Ibsp* were similarly expressed in both cell lines (Fig. 4B). Additional Wnt3a stimulation led to a further reduction in the mRNA level of chondrocyte-specific genes regardless of the genotype (Fig. 4C,D). On the other hand, *Runx2* mRNA expression levels did not change upon activation of canonical Wnt signaling, whereas *Atf4* mRNA expression decreased by approximately 35% in control and *β-catenin* knockdown cells. Interestingly, *Sp7* transcripts increased slightly in treated control cells (Fig. 4C), but did not change in knockdown cells upon Wnt3a treatment (Fig. 4D). *Ibsp* was the only gene, whose mRNA expression level strongly increased in both cell lines upon Wnt3a stimulation. In aggregate, *β-catenin* knockdown primary chondrocytes already had a more bone-specific basic expression profile before Wnt3a stimulation compared to control primary chondrocytes. However, Wnt3a treatment almost completely abrogated chondrocyte-specific gene expression in both cell lines and induced the expression of the osteoblast-specific extracellular matrix component *Ibsp*. This shows that despite reduced *β-catenin* levels, knockdown cells are still able to respond normally to canonical Wnt signaling.

## DISCUSSION

Reducing β-catenin expression to 25% has profound effects on the development of the limbs, spine and tail. Given the multiple roles of canonical Wnt signaling in the formation, patterning and maintenance of bones (Capdevila and Izpisúa Belmonte, 2001; Duan and Bonewald, 2016), our approach helps to identify the morphogenetic processes that can proceed normally even at low β-catenin levels, in contrast to those that require high β-catenin dosages.

### Defects due to impaired outgrowth and induction

The most striking morphological findings in our mice were the absence of hindlimbs and the shortened, crooked tail, most likely resulting from impaired outgrowth of these structures. A human genetic syndrome that is characterized by the absence of all four limbs (Tetra-amelia syndrome) results from the autosomal recessive inactivation of the *WNT3* gene (Niemann et al., 2004). In mice, *Wnt3a* knockout leads to the absence of hindlimbs, deformed forelimbs and misshaped tails (Takada et al., 1994). Furthermore, in *Wnt5a* knockout mice the outgrowth of limbs and tail is severely compromised (Yamaguchi et al., 1999). However, whereas the loss of Wnt3a or Wnt5a produces a very specific upstream block of canonical Wnt signaling at the level of ligand-receptor interaction, the phenotype of our mice results from inadequate transduction of all canonical Wnt signals at the level of target gene transactivation. In more detail, Wnt3 is centrally involved in the establishment and maintenance of the AER via the activation of the Wnt target gene *Fgf8* (Barrow et al., 2003; Kengaku et al., 1998; Narita et al., 2007). The AER is the master organizer of the growing limb, regulating the generation and allocation of progenitor cells to the future limb bones. Loss of AER function leads to impaired proliferation of progenitor cells and thus the premature termination of limb development (Lu et al., 2008). We previously showed that reduced β-catenin expression leads to diminished *Fgf8* expression levels in the tailbud (Rudloff and Kemler, 2012). Moreover, correct Fgf expression is required for the maintenance of a stem cell population of mesodermal cells that contributes to the growth of the embryo (Boulet and Capecchi, 2012). Of note, the teratogenic effect of thalidomide, which leads to deformed limbs, is caused by the untimely inhibition of canonical Wnt signaling and increased cell death (Knobloch et al., 2007). Therefore, the proper induction and outgrowth of tail and limbs not only depends on the integrity of the Wnt-Fgf axis, but also on the expression level of β-catenin. As shown in this study, 25% β-catenin expression levels are not sufficient for the maintenance of this signaling axis, probably due to the early exhaustion of the proliferation potential of progenitor cells. This also offers an explanation as to why hindlimbs show a higher degree of distortion than forelimbs. Since the cells in the caudal embryo have passed through more cells divisions before they become part of the future hindlimb, they might not anymore be able to generate enough cells to form a limb.

### Defects due to impaired patterning

Although forelimbs are present in *β-catenin* knockdown embryos, their structure is abnormal; the autopodia, and rarely the zeugopodia, also show irregularities. In the forelimbs the phenotype is highly variable, ranging from the addition of multiple irregularly shaped digits within the plane of the paw and protruding the paw at anomalous angles, to reduced numbers of phalanges or the absence of one of the two zeugopodial bones. Specification of the anterior-posterior identity of the bones of zeugopodium and autopodium is achieved by a Sonic hedgehog (Shh) morphogen gradient, originating from the zone of polarizing activity (ZPA), located at the posterior end of the growing limb (Capdevila and Izpisúa Belmonte, 2001; Chiang et al., 2001). Canonical Wnt signaling has also been shown to be important for the maintenance of the ZPA in concert with the AER (Pearse and Tabin, 1998). Hereby, Fgfs from the AER form a positive feedback loop involving Shh and the Bmp inhibitor Gremlin1, which in turn sustain the β-catenin activation required for the maintenance of the AER. The coordinated activity of ZPA and AER is further underlined by common enhancers that regulate the expression of important patterning genes from both signaling centers (VanderMeer et al., 2014). In our *β-catenin* knockdown embryos all members involved in the feed forward loop between AER and ZPA are diminished. Thus, our findings do not allow us to draw definite conclusions on whether the patterning activity of the ZPA functions normally with reduced *β-catenin* levels, since the AER defects might obscure ZPA-dependent phenotypes. On the other hand, the observed patterning defects might arise from small local differences in Shh expression. A more specific knockdown of β-catenin in the AER or ZPA is needed to clarify this question.

### Differentiation of cartilage and bone

There are several steps during skeletogenesis where Wnt/β-catenin signaling has an inhibiting effect, while at others activating effects predominate. Early in osteogenesis, cells forming the skeletal primordia differentiate into chondrocytes or osteoblast precursors. In this process, canonical Wnt signaling directly promotes the generation of osteoblast precursor cells, marked by Runx2 and Col2a1 (Regard et al., 2012). In contrast, blocking Wnt signaling at this step favors the differentiation into Sox9-positive chondrocytes (Bennett et al., 2005; Huang et al., 2007). Osteoblast precursors themselves are prevented from differentiation into chondrocytes by canonical Wnt signaling. Conditional bone-specific loss of *β-catenin* during early development has been shown to lead to abnormalities in osteoblastogenesis and ectopic development of cartilage (Day et al., 2005; Hill et al., 2005). The primary chondrocytes isolated from *β-catenin* knockdown embryos in our study showed a *β-catenin* expression level of approximately 25% compared to wild-type cells. This corresponds exactly to the expected expression level (Rudloff and Kemler, 2012) and confirms the usability of our mouse model. Based on the literature, we expected that the knockdown of *β-catenin* would also lead to increased cartilage differentiation and a decreased number of osteoblasts, and subsequently a reduction in calcified bone. However, to our surprise, the histomorphometrical analysis of forelimb zeugopodia showed no significant difference in the mineralized bone area between wild-type and mutant embryos. Moreover, the expression of cartilage-specific genes was lower in primary *β-catenin* knockdown cells than in control cells. These findings suggest that *β-catenin* knockdown does not favor the formation of additional cartilage. In agreement with this, canonical Wnt signaling was also described to have an inhibitory effect on the transition from early osteoblasts to mature osteoblasts. Therefore, an early potential developmental bias towards cartilage differentiation in our knockdown embryos could be counterbalanced by an accelerated maturation of osteoblasts at later stages. Furthermore, stimulation of canonical Wnt signaling almost completely abolished the expression of cartilage markers and significantly increased the expression of the bone-specific matrix protein *Ibsp* in both cell types. Based on these findings our isolated cells most likely correspond to a mix of chondrocytes and osteoblast precursors, which, after Wnt3a treatment, mature into Ibsp-positive early osteoblasts (Regard et al., 2012). Moreover, 25% of β-catenin expression seems to be sufficient to sustain normal mineralization of bone.

### Conclusions and outlook

Our study demonstrates that β-catenin expression levels as low as 25% are sufficient for the correct differentiation of cartilage and bone in skeletal primordia, but not sufficient for the correct patterning of limbs and tail (summarized in Table 1). Thus, the phenotype of our mutant mice most likely results from incorrectly specified signaling centers such as AER and ZPA together with a reduced proliferative capacity of skeletal progenitor cells, but not from differentiation defects of chondrocytes and osteoblasts. Our findings also shed more light on β-catenin dependent processes during bone regeneration and fracture repair. During bone repair, similar mechanisms are at work as during embryonic development. Here, mutations increasing β-catenin levels show an accelerated repair process (Arioka et al., 2013), but also lead to unwanted side effects such as higher bone mass or the formation of excess fibrous tissue as in pseudarthrosis or osteoarthritis (Ghadakzadeh et al., 2016; Tornero-Esteban et al., 2015). Conversely, loss of canonical Wnt signaling impairs the fracture healing process (Burgers et al., 2016; Huang et al., 2012). Since the level of Wnt proteins declines with age (Rauner et al., 2008), treatment of low bone mass in elderly patients with novel Wnt agonists such as antibodies against Sclerostin or Dickkopf will become more and more important (Florio et al., 2016; Ke et al., 2012). However, how and to what extent β-catenin levels change with increasing age remains to be elucidated. From our study, we hypothesize that 25% is still sufficient to sustain bone formation and repair. In future studies, markers for bone turnover and the bone microstructure will be assessed in greater detail to gain more insight on the health status of bone in conditions of reduced β-catenin expression.

**Table 1.**
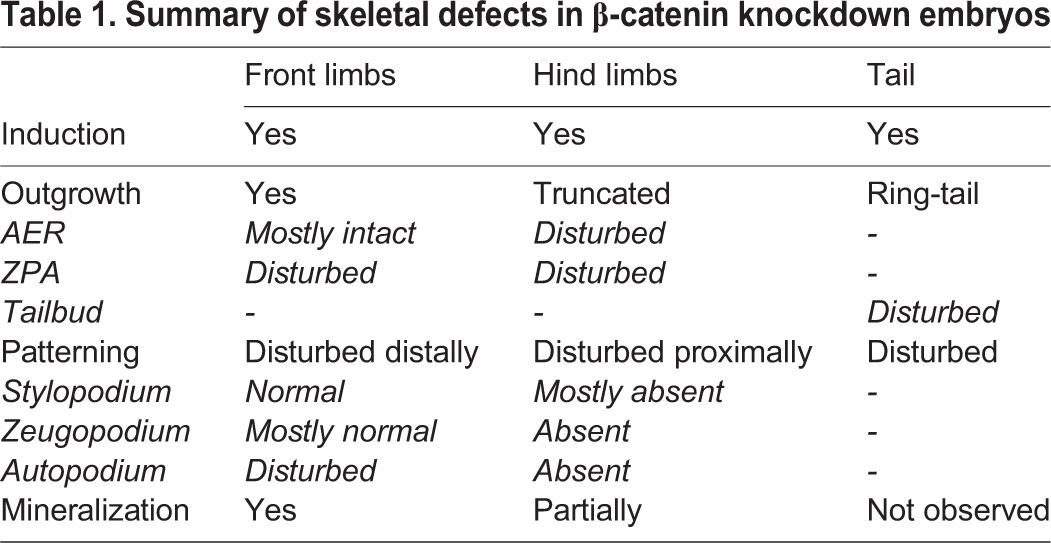
**Summary of skeletal defects in β-catenin knockdown embryos**

## MATERIAL AND METHODS

### Animal models

The experimental procedures were approved by the committee for animal experimentation of the canton Bern. All procedures involving experimental animals were performed in compliance with local animal welfare laws, guidelines and policies. The following genetic traits were combined on a C57BL/6*129S6/SvEvTac background: (1) Cre-recombinase under the control of the caudal type homeobox promoter (*Cdx1::Cre*) (Hierholzer and Kemler, 2009); (2) a conditional β-catenin allele, in which exons 2-6 are flanked by loxP sites (*β-catenin flox/flox*) (Brault et al., 2001); (3) β-catenin under control of the ROSA26 promotor (*ROSA26::β-catenin*) (Rudloff and Kemler, 2012). In (*Cdx1::Cre*)(*β-catenin flox/flox*)(*ROSA26::β-catenin*) mice, the endogenous *β-catenin* will be deleted in the caudal half of the embryo and replaced by the *ROSA26* transgene. Mice that express *β-*catenin from both ROSA26 loci retain approximately 25% of β-catenin compared to wild types. In this study, embryos of both sexes were analyzed at E17.5 and E18.5. Genotyping of endogenous *β-catenin* was performed as previously described (Brault et al., 2001).

### Histology, skeletal staining and micro CT imaging

For Hematoxylin-Eosin (HE) staining, paraffin embedded tissues were cut into 7 μm sections and rehydrated. Sections were then incubated in 1:10 dilution of Harris hematoxylin solution modified (Sigma-Aldrich, Buchs, Switzerland) for 3 min. After washing the slide in tap water for 10 min, they were incubated in Eosin Y solution alcoholic (Sigma-Aldrich, Buchs, Switzerland) for 2 min, followed by dehydration. Finally, the slides were mounted with Eukitt mounting solution (Grogg Chemie, Stettlen, Switzerland). For von-Kossa staining for mineralized bone, the rehydrated sections were incubated in 1% silver nitrate (Merck, Germany) solution under UV-light for 3 h, rinsed in several changes of deionized water and incubated in 5% sodium thiosulfate (Merck, Germany) for 5 min to remove unreacted silver nitrate. After counter-staining with Nuclear Fast Red for 5 min, the dehydrated slides were mounted. For whole mount skeletal staining, Alcian Blue and Alzarin Red (Sigma-Aldrich, Buchs, Switzerland) staining was used as previously described (Rudloff and Kemler, 2012). For quantitative analysis, the slides were digitalized using a Nikon DS-Ri1 camera. Digital images were sent to Morphisto Evolutionsforschung und Anwendung GmbH (Frankfurt am Main, Germany) for histological and histomorphological analysis using the Histoquest 3.0 software (TissueGnostics, Vienna, Austria). Micro CT scans were made using a mCT 40 (Scanco Medical AG, Brüttisellen, Switzerland) with the following parameters: X-ray source (55 kVp with 145 mA at medium resolution), diameter of sample holder (16 mm), integration time (300 ms), measurement time (2 h). The evaluation of the reconstructed 2D Images (total 1600 slices, 25.6 mm length) was made with a 3D segmentation of volume of interest script from Scanco Medical AG.

### Isolation and culture of primary chondrocytes

Mouse rib chondrocytes were isolated from E18.5 embryos as described (Thirion and Berenbaum, 2004). Briefly, ventral ribcages were dissected in a petri dish, washed several times with sterile PBS and incubated in 3 mg/ml collagenase D at 37°C for 90 min. The cartilage pieces were freed from all soft tissue and incubated in DMEM supplemented with 2 mM L-glutamine, 50 U/ml penicillin, 0.05 mg/ml streptomycin, 10% FCS and 3 mg/ml collagenase D until single cells were visible. After two washing steps in PBS, the cells were seeded at 10^5^ cells/cm^2^. Before isolation of RNA some cells were stimulated with 100 ng/ml Wnt3a (Peprotech, UK).

### RNA isolation and qRT-PCR

Total RNA was isolated with TRIzol^®^ reagent according to the manufacturer's protocol. RNA concentration and quality was determined with a Nanodrop 1000 spectrophotometer (Thermo Fisher Scientific, Switzerland) and 500-1000 ng were transcribed into cDNA using the Fast transcriptor cDNA synthesis kit (Roche, Switzerland). For subsequent steps the cDNA was diluted to 2 ng/µl. All qRT-PCR reactions were run in triplicate using the Universal Probe Library system from Roche. 9 µl cDNA and the appropriate primers and probes (see Table S1) were used for each reaction.

### Statistical analysis

The results of qRT-PCR and von-Kossa stained area were tested for significance using unpaired, two-tailed Student's *t*-tests. *P*-values <0.05 were considered statistically significant.
